# Supplementary material for: Modest google trends associations with mental health patient and general population deaths by suicide: A time-series analysis using patient-provided search terms
Source: PLoS One. 2026 Apr 15;21(4):e0344926. doi: 10.1371/journal.pone.0344926 (PMC13082702; doi:10.1371/journal.pone.0344926)
Supplement: S1 File — (DOCX) [file pone.0344926.s001.docx]

S1 File. Comparison between participants who did and those who did not provide search terms.

**Table 1.** Sociodemographic characteristics of suicidal mental health patients who engaged in SRIU who did and did not provide the search terms they used to search for suicide-related content.

|  | Provided search terms (N=196) | Did not provide search terms (N=337) | Total (N=533) | Chi-squared test | p-value |
| --- | --- | --- | --- | --- | --- |
| **Age groups** |  |  |  |  |  |
| 18-24 | 45 (23.0%) | 79 (23.4%) | 124 (23.3%) | <0.001 | 0.983 |
| 25-34 | 84 (42.9%) | 158 (46.9%) | 242 (45.4%) | 0.656 | 0.418 |
| 35-44 | 40 (20.4%) | 67 (19.9%) | 107 (20.1%) | 0.001 | 0.973 |
| 45-54 | 23 (11.7%) | 20 (5.9%) | 43 (8.1%) | 4.866 | 0.027 |
| 55-74 | 4 (2.0%) | 13 (3.9%) | 17 (3.2%) | 0.802 | 0.371 |
| **Gender** |  |  |  |  |  |
| Female | 133 (67.9%) | 211 (62.6%) | 344 (64.5%) | 1.270 | 0.260 |
| Male | 51 (26.0%) | 99 (29.4%) | 150 (28.1%) | 0.534 | 0.465 |
| Other/Prefer not to say | 12 (6.1%) | 27 (8.0%) | 39 (7.3%) | 0.403 | 0.525 |
| **Gender matching birth sex** |  |  |  |  |  |
| Yes | 170 (86.7%) | 275 (81.6%) | 445 (83.5%) | 2.010 | 0.156 |
| No, I am non-binary | 12 (6.1%) | 35 (10.4%) | 47 (8.8%) | 2.296 | 0.130 |
| No, I am transgender | 11 (5.6%) | 19 (5.6%) | 30 (5.6%) | <0.001 | 0.999 |
| Other/Prefer not to say | 3 (1.5%) | 8 (2.4%) | 11 (2.1%) | 0.119 | 0.731 |
| **Ethnicity** |  |  |  |  |  |
| Asian/Asian British | 5 (2.6%) | 12 (3.6%) | 17 (3.2%) | 0.148 | 0.701 |
| Black/African/Caribbean/Black British | 5 (2.6%) | 1 (0.3%) | 6 (1.1%) | 3.814 | 0.051 |
| Mixed/multiple ethnic group | 15 (7.7%) | 16 (4.7%) | 31 (5.8%) | 1.416 | 0.234 |
| White/White British/White Irish | 164 (83.7%) | 301 (89.3%) | 465 (87.2%) | 3.058 | 0.080 |
| Other/Prefer not to say^a^ | 7 (3.6%) | 7 (2.1%) | 14 (2.6%) | 0.577 | 0.448 |
| **Sexual orientation** |  |  |  |  |  |
| Lesbian/Gay/Bisexual | 72 (36.7%) | 128 (38.0%) | 200 (37.5%) | 0.038 | 0.846 |
| Heterosexual | 83 (42.3%) | 138 (40.9%) | 221 (41.5%) | 0.050 | 0.822 |
| Other/Prefer not to say | 41 (20.9%) | 71 (21.1%) | 112 (21.0%) | <0.001 | 0.999 |
| **Highest level of education** |  |  |  |  |  |
| A or AS Levels / Highers or Advanced Highers and less | 52 (26.5%) | 115 (34.1%) | 167 (31.3%) | 2.978 | 0.084 |
| University degree and above | 121 (61.7%) | 176 (52.2%) | 297 (55.7%) | 4.165 | 0.041 |
| Other/Prefer not to say^b^ | 23 (11.7%) | 46 (13.6%) | 69 (12.9%) | 0.251 | 0.616 |
| **Employment status** |  |  |  |  |  |
| In paid employment (including part-time, self-employed) | 105 (53.6%) | 164 (48.7%) | 269 (50.5%) | 1.005 | 0.316 |
| Unemployed | 33 (16.8%) | 69 (20.5%) | 102 (19.1%) | 0.838 | 0.360 |
| Full-time student | 18 (9.2%) | 32 (9.5%) | 50 (9.4%) | <0.001 | 0.999 |
| On long-term sick leave | 28 (14.3%) | 49 (14.5%) | 77 (14.4%) | <0.001 | 0.999 |
| Other/Prefer not to say | 12 (6.1%) | 23 (6.8%) | 35 (6.6%) | 0.018 | 0.893 |
| **Relationship status** |  |  |  |  |  |
| Single | 108 (55.1%) | 153 (45.4%) | 261 (49.0%) | 0.684 | 0.408 |
| In a relationship (not co-habiting) | 19 (9.7%) | 42 (12.5%) | 61 (11.4%) | 4.287 | 0.038 |
| Married / co-habiting / civil partnership | 58 (29.6%) | 112 (33.2%) | 170 (31.9%) | 0.599 | 0.439 |
| Separated / divorced | 6 (3.1%) | 17 (5.0%) | 23 (4.3%) | 0.749 | 0.387 |
| Other/Prefer not to say | 5 (2.6%) | 13 (3.9%) | 18 (3.4%) | 0.310 | 0.578 |
| ^a^includes asexual, pansexual, and questioning; ^b^includes homemaker, training scheme, and retired; | | | | | |

**Table 2.** Clinical and mental health care characteristics of suicidal mental health patients who engaged in SRIU who did and did not provide the search terms they used to search for suicide-related content.

|  | Provided search terms (N=196) | Did not provide search terms (N=337) | Total (N=533) | Chi-squared/ Wilcoxon test | p-value |
| --- | --- | --- | --- | --- | --- |
| **Psychiatric diagnosis** |  |  |  |  |  |
| No diagnosis | 38 (19.4%) | 73 (21.7%) | 111 (20.8%) | 0.305 | 0.581 |
| Schizophrenia and other delusional disorders | 14 (7.1%) | 28 (8.3%) | 42 (7.9%) | 0.141 | 0.707 |
| Affective disorder | 139 (70.9%) | 247 (73.3%) | 386 (72.4%) | 0.750 | 0.386 |
| Anxiety/Phobia/OCD^a^ | 113 (57.7%) | 192 (57.0%) | 305 (57.2%) | <0.001 | 0.999 |
| PTSD^b^ | 66 (33.7%) | 128 (38.0%) | 194 (36.4%) | 1.143 | 0.285 |
| Eating disorder | 43 (21.9%) | 77 (22.8%) | 120 (22.5%) | 0.062 | 0.804 |
| Alcohol/drug dependence | 38 (19.4%) | 53 (15.7%) | 91 (17.1%) | 0.756 | 0.384 |
| Personality disorder | 59 (30.1%) | 122 (36.2%) | 181 (34.0%) | 2.244 | 0.134 |
| Autism spectrum disorder | 46 (23.5%) | 92 (27.3%) | 138 (25.9%) | 0.995 | 0.318 |
| ADHD^c^ | 30 (15.3%) | 63 (18.7%) | 93 (17.4%) | 0.946 | 0.331 |
| Other | 32 (16.3%) | 57 (16.9%) | 89 (16.7%) | 0.022 | 0.882 |
| **Mental health care** |  |  |  |  |  |
| In-patient treatment in the last 12 months | 26 (13.3%) | 69 (20.5%) | 95 (17.8%) | 3.842 | 0.050 |
| Prescribed psychotropic medication in the last 12 months | 158 (80.6%) | 271 (80.4%) | 429 (80.5%) | <0.001 | 0.999 |
| Satisfaction with mental health service provision | 4.00 (2–6.57) | 4.00 (2–6.00) | 4.00 (2–6.30) | 30000.5 | 0.741 |

^a^obsessive-compulsive disorder; ^b^post-traumatic stress disorder; ^c^attention-deficit/hyperactivity disorder

**Table 3.** Suicidal thoughts of suicidal mental health patients who engaged in SRIU who did and did not provide the search terms they used to search for suicide-related content.

|  | Provided search terms (N=196) | Did not provide search terms (N=337) | Total (N=533) | Chi-squared/ Wilcoxon test | p-value |
| --- | --- | --- | --- | --- | --- |
| **Have you experienced suicidal thoughts in the last 12 months?** |  |  |  |  |  |
| Yes | 192 (98.0%) | 329 (97.6%) | 521 (97.7%) | <0.001 | 0.999 |
| Prefer not to say | 4 (2.0%) | 8 (2.4%) | 12 (2.3%) |  |  |
| **How often did you experience suicidal thoughts?** |  |  |  |  |  |
| Just once | 2 (1.0%) | 6 (1.8%) | 8 (1.5%) | 0.115 | 0.734 |
| Every few months | 26 (13.3%) | 33 (9.8%) | 59 (11.1%) | 1.107 | 0.293 |
| Once a month | 10 (5.1%) | 28 (8.3%) | 38 (7.1%) | 1.542 | 0.214 |
| Once a week | 28 (14.3%) | 44 (13.1%) | 72 (13.5%) | 0.052 | 0.820 |
| 2-5 times a week | 48 (24.5%) | 110 (32.6%) | 158 (29.6%) | 3.866 | 0.049 |
| Every day or almost every day | 78 (39.8%) | 106 (31.5%) | 184 (34.5%) | 3.213 | 0.073 |
| **What was the intensity of your suicidal thoughts?** | 7.00 (5–8.00) | 6.50 (5–7.70) | 6.60 (5–7.77) | 32800 | 0.360 |
| **Did you disclose your thoughts of suicide to anyone?** |  |  |  |  |  |
| Did not disclose to anyone | 17 (8.7%) | 34 (10.1%) | 51 (9.6%) | 0.165 | 0.684 |
| Disclosed to a friend/family member | 65 (33.2%) | 111 (32.9%) | 176 (33.0%) | <0.001 | 0.999 |
| Disclosed to a mental health professional | 141 (71.9%) | 233 (69.1%) | 374 (70.2%) | 0.237 | 0.626 |
| **Have you attempted suicide in the last 12 months?** |  |  |  |  |  |
| Yes | 65 (33.2%) | 106 (31.5%) | 171 (32.1%) | 0.097 | 0.755 |
| No/Prefer not to say | 131 (66.8%) | 231 (68.5%) | 362 (67.9%) |  |  |
| **How much planning was involved in your suicide attempt?** | 7.70 (5–8.95) | 6.00 (4–8.00) | 6.75 (4–8.30) | 4080 | 0.002 |
| **How many times have you attempted suicide in the last 12 months?** |  |  |  |  |  |
| Once | 24 (12.2%) | 45 (13.4%) | 69 (12.9%) | 0.430 | 0.512 |
| Twice | 22 (11.2%) | 31 (9.2%) | 53 (9.9%) | 0.144 | 0.704 |
| Three or more times | 19 (9.7%) | 28 (8.3%) | 47 (8.8%) | 0.022 | 0.881 |
| **Hospitalised due to suicide attempt** |  |  |  |  |  |
| Yes | 42 (21.4%) | 61 (18.1%) | 103 (19.3%) | 0.544 | 0.461 |
| No | 22 (11.2%) | 43 (12.8%) | 65 (12.2%) |  |  |
| **Did you disclose plans to attempt to anyone?** |  |  |  |  |  |
| Did not disclose to anyone | 35 (17.9%) | 66 (19.6%) | 101 (18.9%) | 0.293 | 0.588 |
| Disclosed to a friend/family member | 6 (3.1%) | 12 (3.6%) | 18 (3.4%) | 0.003 | 0.955 |
| Disclosed to mental health professional | 18 (9.2%) | 25 (7.4%) | 43 (8.1%) | 0.405 | 0.525 |

**Table 4.** Suicide-related internet use of mental health patients who did and did not provide the search terms they used to search for suicide-related content.

|  | Provided search terms (N=196) | Did not provide search terms (N=337) | Total (N=533) | Chi-squared/ Wilcoxon test | p-value |
| --- | --- | --- | --- | --- | --- |
| **Type of SRIU** |  |  |  |  |  |
| Searched for suicide related content/information | 174 (88.8%) | 282 (83.7%) | 456 (85.6%) | 2.208 | 0.137 |
| Created suicide related content/information | 25 (12.8%) | 41 (12.2%) | 66 (12.4%) | 0.004 | 0.950 |
| Engaged with (commented/reposted) suicide-related content | 57 (29.1%) | 116 (34.4%) | 173 (32.5%) | 1.377 | 0.241 |
| Accidentally came across suicide-related content | 62 (31.6%) | 114 (33.8%) | 176 (33.0%) | 0.180 | 0.671 |
| Used internet to interact/connect with others | 99 (50.5%) | 172 (51.0%) | 271 (50.8%) | 0.001 | 0.978 |
| **Motivation for SRIU** |  |  |  |  |  |
| Seeking information on support/help | 107 (54.6%) | 197 (58.5%) | 304 (57.0%) | 0.606 | 0.436 |
| Seeking information on methods of suicide | 136 (69.4%) | 182 (54.0%) | 318 (59.7%) | 11.552 | 0.001 |
| Seeking support from friends/peers | 48 (24.5%) | 98 (29.1%) | 146 (27.4%) | 1.092 | 0.296 |
| Interacting with celebrity bloggers/influencers | 3 (1.5%) | 15 (4.5%) | 18 (3.4%) | 2.406 | 0.121 |
| Engaging in discussions about suicide/harming oneself | 46 (23.5%) | 72 (21.4%) | 118 (22.1%) | 0.208 | 0.648 |
| Sharing personal experiences of suicidality | 57 (29.1%) | 71 (21.1%) | 128 (24.0%) | 3.933 | 0.047 |
| Researching medication | 102 (52.0%) | 162 (48.1%) | 264 (49.5%) | 0.630 | 0.427 |
| Curiosity about suicide | 18 (9.2%) | 40 (11.9%) | 58 (10.9%) | 0.666 | 0.415 |
| Raising awareness for suicide prevention | 18 (9.2%) | 38 (11.3%) | 56 (10.5%) | 0.376 | 0.540 |
| Other | 3 (1.5%) | 7 (2.1%) | 10 (1.9%) | 0.014 | 0.907 |
| **Characteristics of SRIU** |  |  |  |  |  |
| Have you searched for a specific site? | 51 (26.0%) | 53 (15.7%) | 104 (19.5%) | 5.662 | 0.017 |
| Do you know where to find information on how to use internet safely when engaging in SRIU? | 81 (41.3%) | 162 (48.1%) | 243 (45.6%) | 5.019 | 0.025 |
| **How often did you engage in SRIU?** |  |  |  |  |  |
| Just once | 15 (7.7%) | 26 (7.7%) | 41 (7.7%) | 0.005 | 0.945 |
| Every few months | 50 (25.5%) | 86 (25.5%) | 136 (25.5%) | 0.109 | 0.741 |
| Once a month | 43 (21.9%) | 72 (21.4%) | 115 (21.6%) | 0.015 | 0.903 |
| Once a week | 37 (18.9%) | 66 (19.6%) | 103 (19.3%) | 0.198 | 0.657 |
| 2-5 times a week | 38 (19.4%) | 49 (14.5%) | 87 (16.3%) | 1.020 | 0.313 |
| Every day or almost every day | 12 (6.1%) | 15 (4.5%) | 27 (5.1%) | 0.221 | 0.638 |
| How helpful or harmful was your SRIU?  (0 helpful, 10 harmful) | 5.00 (4–6.40) | 5.00 (4–6.00) | 5.00 (4–6.10) | 31498.5 | 0.379 |
| **Online suicide prevention** |  |  |  |  |  |
| Have you seen any online suicide prevention interventions? | 171 (87.2%) | 262 (77.7%) | 433 (81.2%) | 1.425 | 0.233 |
| If yes, have you engaged with them? | 83 (42.3%) | 113 (33.5%) | 196 (36.8%) | 0.663 | 0.415 |
| If you interacted with the interventions, did you find them helpful?  (0 helpful, 10 harmful) | 5.00 (4–6.50) | 5.00 (4–7.00) | 5.00 (4–6.90) | 12488 | 0.187 |
| **Enquiry about SRIU** |  |  |  |  |  |
| Have you been asked about SRIU by your mental health professional? | 23 (11.7%) | 37 (11.0%) | 60 (11.3%) | <0.001 | 0.999 |
| Have you told your mental health professional about your SRIU? | 78 (39.8%) | 92 (27.3%) | 170 (31.9%) | 5.033 | 0.025 |

SRIU=suicide-related internet use
