# Supplementary material for: Modest google trends associations with mental health patient and general population deaths by suicide: A time-series analysis using patient-provided search terms
Source: PLoS One. 2026 Apr 15;21(4):e0344926. doi: 10.1371/journal.pone.0344926 (PMC13082702; doi:10.1371/journal.pone.0344926)
Supplement: Attachment — Submitted filename: Response to Reviewers PONE-D-25-53803.docx [file pone.0344926.s003.docx]

PONE-D-25-53803

Modest Google Trends Associations with Mental Health Patient and General Population Deaths by Suicide: A Time-Series Analysis Using Patient-Provided Search Terms

Dear Dr Mitra,

Thank you for your interest in the paper and the helpful comments from the reviewers. We have now responded to the comments which we believe has improved the paper, and revisions are highlighted in yellow throughout. The journal requirements have now been addressed and we look forward to hearing from you.

Kind regards,

Lana Bojanić (on behalf of all authors)

Journal Requirements:

When submitting your revision, we need you to address these additional requirements.

1. Please ensure that your manuscript meets PLOS ONE's style requirements, including those for file naming. The PLOS ONE style templates can be found at

[https://journals.plos.org/plosone/s/file?id=wjVg/PLOSOne_formatting_sample_main_body.pdf [journals.plos.org]](https://urldefense.com/v3/__https:/journals.plos.org/plosone/s/file?id=wjVg*PLOSOne_formatting_sample_main_body.pdf__;Lw!!PDiH4ENfjr2_Jw!ETS4byWIRinceKvsIqUEoQY8MqyN9rIZW6VnxZS51ZcNe7aWN83CaLX1nJvdjkl5odJwvhMopcyhchDuh1DdBfp1GFS7$) and [https://journals.plos.org/plosone/s/file?id=ba62/PLOSOne_formatting_sample_title_authors_affiliations.pdf [track.editorialmanager.com]](https://urldefense.com/v3/__https:/track.editorialmanager.com/CL0/https:*2F*2Fjournals.plos.org*2Fplosone*2Fs*2Ffile*3Fid=ba62*2FPLOSOne_formatting_sample_title_authors_affiliations.pdf/1/010f019b5a00be90-d2350028-8275-47b9-91a1-f5dde4c44a6f-000000/XH0k6iSBRlFwY4BikHjjMRPYmJWtcRuUwbPXaVENcxE=243__;JSUlJSUlJQ!!PDiH4ENfjr2_Jw!ETS4byWIRinceKvsIqUEoQY8MqyN9rIZW6VnxZS51ZcNe7aWN83CaLX1nJvdjkl5odJwvhMopcyhchDuh1DdBZ7K4oCt$)

Response: The manuscript has now been reformatted to meet the journal’s style requirements.

2. In your Methods section, please include additional information about your dataset and ensure that you have included a statement specifying whether the collection and analysis method complied with the terms and conditions for the source of the data.

Response: The following has now been included at the end of methods section:

“Data availability and compliance

This study analysed three data sources, each with distinct availability and terms:

(i) Participant-generated search terms: Participants who provided search terms were recruited online as part of a cross-sectional anonymous survey on suicide-related internet use of mental health patients between June 1 and December 31, 2023. They provided written informed consent by ticking the box in the online questionnaire hosted on Qualtrics. The search terms and coded categories are available upon request from the corresponding author LB (lana.bojanic-2@manchester.ac.uk). Due to ethical considerations regarding the public dissemination of potentially harmful search terms related to suicidality (24), the full list of search terms is not included in the main text.

(ii) Google Trends data: These time-series data are publicly available via Google Trends (https://trends.google.com) and can be reproduced using the methodology described in the Methods section.

(iii) Suicide mortality data: These data are governed by strict confidentiality regulations and are not available for public distribution. Access to the Office for National Statistics and National Records of Scotland data is subject to request. For more information, see <https://www.ons.gov.uk/aboutus/whatwedo/statistics/requestingstatistics> (ONS) and <https://www.researchdata.scot/accessing-data/begin-an-enquiry> (NRS). Access to mental health patient suicide data can be requested via application to the Healthcare Quality Improvement Partnership (<https://www.hqip.org.uk/national-programmes/accessing-ncapop-data/>).

All authors had full access to all of the data in the study and take responsibility for the integrity of the data and the accuracy of data analysis. The collection and analysis method complied with the terms and conditions of the source of data.”

3. In the online submission form, you indicated that [Since the data in question represents search terms used for suicide-related purposes, the data will be available to other researchers by request only.].

All PLOS journals now require all data underlying the findings described in their manuscript to be freely available to other researchers, either 1. In a public repository, 2. Within the manuscript itself, or 3. Uploaded as supplementary information.

This policy applies to all data except where public deposition would breach compliance with the protocol approved by your research ethics board. If your data cannot be made publicly available for ethical or legal reasons (e.g., public availability would compromise patient privacy), please explain your reasons on resubmission and your exemption request will be escalated for approval.

Response: Search term data: The raw participant-level data for this study cannot be made publicly available. Our ethics approval from the University of Manchester Research Ethics Committee 1 (ref: 2023-16133-28055) explicitly requires that:

•	Data may only be accessed and analysed within the NCISH office and are stored on an isolated network not connected to general university infrastructure.

•	Access is restricted to the named research team (LB, IH, SI, SF, PT) involved in this study.

These restrictions were imposed as a condition of the ethical approval due to the sensitive nature of the data (mental health patients with suicidal thoughts/behaviours). Making these data publicly available would breach both our approved protocol and UK GDPR/Data Protection Act 2018 obligations. Data requests and inquiries may be sent to: research.ethics@manchester.ac.uk.

The search volume data is fully dependent on the participant-generated search term data; without the exact search terms, exact Google Trends data can not be replicated. Therefore, providing access to it would not enable full replication of our analysis. Replication would require access to the search term dataset, which is restricted under our ethics approval.

Suicide mortality data: Suicide mortality contain potentially identifying and, in case of patient suicide data, sensitive patient information. These data are governed by strict confidentiality regulations and are not available for public distribution. Further, these data are owned by third party organisations. Access to the Office for National Statistics and National Records of Scotland data is subject to request. For more information, see https://www.ons.gov.uk/aboutus/whatwedo/statistics/requestingstatistics (ONS) and

https://www.researchdata.scot/accessing-data/begin-an-enquiry (NRS). Access to mental health patient suicide data can be requested via application to the Healthcare Quality Improvement Partnership (https://www.hqip.org.uk/national-programmes/accessing-ncapop-data/).

Therefore, we request an exemption from public data deposition for these datasets.

4. Please note that you should ensure that each author is linked to an affiliation, and clearly indicate the corresponding author, as per our author instructions. Authors’ affiliations should reflect the institution where the work was done (if authors moved subsequently, you can also list the new affiliation stating “current affiliation:….” as necessary).

Response: I confirm that all authors are linked to an affiliation.

5. Please include your full ethics statement in the ‘Methods’ section of your manuscript file, and please make sure that the information provided in your submission is also the same as what will be included in the manuscript. In your statement, please include the full name of the IRB or ethics committee who approved or waived your study, as well as whether or not you obtained informed written or verbal consent. If consent was waived for your study, please include this information in your statement as well.

Response: The full ethical approval section is now as follows:

“Ethical approval

Ethical approval for collecting and analysing participants' suicide-related search terms was obtained from the University of Manchester Research Ethics Committee 1 on the 3rd of April 2023 (ref: 2023-16133-28055). Suicide data is routinely obtained by the National Confidential Inquiry into Suicide and Safety in Mental Health with approval from the North West – GM South REC (reference: ERP/96/136) and Section 251 Approval under the NHS Act 2006 (originally Section 60 of the Health and Social Care Act 2001) (reference: 23/CAG/0024) for England and Wales and Public Benefit and Privacy Panel for Health and Social Care (HSC-PBPP) (reference: 2021-0114) for Scotland.”

Also, we included the following regarding consent in the data availability and compliance section:

“Participants who provided search terms were recruited online as part of a cross-sectional anonymous survey on suicide-related internet use of mental health patients between June 1 and December 31, 2023. They provided written informed consent by ticking the box in the online questionnaire hosted on Qualtrics.”

Review Comments to the Author

Reviewer #1: The paper examines the association between suicide-related Google Trends searches and actual suicide deaths in the UK. The strength of their paper is their use of terms from people with lived experience, which I think is very commendable and a major strength. Overall, their statistical methods for the time series analysis are impeccable, their writing is good, and the care they have taken with the Google Trends data is excellent, although I will point to one major flaw. The results and discussion are thorough but also measured, which is another strength of this paper.
**The Appendix was unfortunately not included in the review file.**
Minor:
P1L21: mental health patients
P4L73-5: Consider changing (1) and (2) so as to distinguish from citation style. Even just 1) and 2), or (a) and (b), etc.

Response: Thank you for your kind comments on the paper’s strengths. The appendix (S1 File) is now included and the minor comments above have been corrected.

Major:
P6L139–143.
I would recommend that the authors reconsider their strategy here:
The Google Trends API data getting started guide1 explain what GT data are:
“The numbers returned are the probability of a short search-session (few consecutive searches), to satisfy the corresponding term restriction, given it was done in the restricted geography (if such exist) and during the time represented by that data point. This probability is multiplied by 10 Million in order to be more human readable. “These values are then scaled to 100 (and rounded) for the Web data.
The values they are working with, are thus the probability of a term being searched, relative to (i.e., weighted by) the nation, for their time period. As such, weighting by the population percentage actually introduces a distortion into the data. I would, however, go further and recommend (but not insist) that the authors consider redoing this work using API data, which provides the raw (unscaled, not-rounded) probability (x 10,000,000 as stated above). This would immensely increase the value of their paper, and allow a much finer comparison of the terms. I have developed and published methods for extracting the required number of samples in a single day, so the main work required would be applying for an API key.

Response: Thank you for this interesting comment. After exploring API data and your work in more detail we recognise the methodological advantages of its consistently scaled data. However, the current API provides access only to a rolling window of approximately 5 years which would not align with our suicide data that spans twelve years. We will nonetheless consider API-based methods in future work.

The values they are working with, are thus the probability of a term being searched, relative to (i.e., weighted by) the nation, for their time period. As such, weighting by the population percentage actually introduces a distortion into the data. Next, it is odd that the authors weighted the GT data by the nation population size, but did not weight the suicide numbers, but used the “Actual monthly numbers of mental health patients who died by suicide” (P6L152). They weighted what they should not have weighted, and did not weight what they should have weighted. However, a further note on weighting. They did not define the time point for the population size (I do note they used population percentage, not population size, P6L140). To properly weight the suicide rates, it would be best to obtain monthly population estimates per nation (or, if those are not available, to estimate them from the nearest available data, such as quarterly or annual population sizes) and express the suicides over time as a rate per 100,000.

Response: Thank you for this detailed comment on weighting methodology.

Regarding weighting GT data, we weighted Google Trends data by population percentage when creating the composite "Great British" measure because: (1) we needed to reflect search behaviour proportionally across the three constituent regions (England, Wales, Scotland) in a way that searches from larger populations have correspondingly greater influence on the composite and (2) this ensured the composite GT index was comparable to the available suicide data. We have clarified in the text that population percentages were determined using yearly population estimates for each year from 2011 to 2022.

We used absolute suicide counts because the suicide data already represented the complete Great Britain population of suicide deaths (both in the general population and in mental health patients). We note that suicide counts and suicide rates are highly correlated (r = 0.97) in our dataset, such that the correlation between the weighted GT composite and absolute suicide counts result in the same statistical inference to what we would obtain using rates. This is further strengthened by our application of Bonferroni correction, which ensured that only robust correlations ‘survive’ multiple testing. Any correlations that would have remained significant under rates would also remain significant under counts, given their near-perfect correlation. We have clarified this in the methods section, under the subheading ‘Mental health patient and general population suicide time-series’.

Reviewer #2: Good work on the study "Modest Google Trends Associations with Mental Health Patient and General Population Deaths by Suicide: A Time-Series Analysis Using Patient-Provided Search Terms". I have attached the suggested review comments to the file attached.

Some Limitations of the study that may be relevant are as follows -

1) Google trend data has measurement limitations- Search volumes are based on sampled rather than complete search activity, and repeated data extractions can yield different values. Although averaging data retrieved on 15 separate days reduced random variation, this approach cannot fully eliminate sampling bias or guarantee full replicability.

Response: Thank you for pointing this out. We have added the following sentence to the strengths and limitations section:

“Averaging Google Trends data collected across 15 different days helped minimise random fluctuations; however, it could not completely remove sampling bias or limits to replicability.”

2) Search behavior doesnt necessarily equate to Suicidal intent ot attempt possibility.- The selected search terms may reflect diverse motivations, including academic interest, media exposure, curiosity, or third-party searching, rather than personal suicidal ideation.

Response: We agree with this comment and the following is included in the conclusion and future directions section of the discussion:

“A recent paper by Rushendran and Chitra (43) states that without knowing who is searching for suicide-related content and why, it is impossible to reliably infer population-level distress or suicide risk from search volumes alone. This study provided some evidence as to who is searching, but the 'why' is still largely unknown. Even seemingly clear 'how to' queries, when considered motivation-wise, may stem from personal crisis, academic research, professional interest, or even curiosity after media coverage.”

3) Weighting Google trends data by population size assumes similar internet access, search behavior, and help-seeking practices across England, Wales, and Scotland. This may mask meaningful regional differences in digital behavior or suicide risk.

Response: Thank you for your comment. Given the broadly similar internet infrastructure across the UK (Ofcom Connected Nations 2025 report), online searching behaviour (Ofcom Online Nations 2025 report), and mental health related help-seeking practices (Wang et al., 2024), we do not expect meaningful regional differences between England, Scotland, and Wales. We have added the following in the Obtaining and preparing Google Trends data subsection of the Methods section: “Weighting Google Trends data by population size was appropriate given the broadly similar internet infrastructure across the UK (16), online searching behaviour (17), and mental health related help-seeking practices (18).”

Ref: <https://www.ofcom.org.uk/siteassets/resources/documents/research-and-data/multi-sector/infrastructure-research/connected-nations-2025/connected-nations-uk-report-2025.pdf?v=407947>

<https://www.ofcom.org.uk/siteassets/resources/documents/research-and-data/online-research/online-nation/2025/online-nations-report-2025.pdf?v=409837>

https://doi.org/10.1136/bmjopen-2023-073731

4) Recent mental health patient suicide data were partially projected due to delays in reporting. While sensitivity analyses restricted to earlier years were conducted, residual bias cannot be ruled out.

Response:: Thank you for your comment. The following sentence in the strengths and limitations section has now been amended to clarify:

“Recent mental health patient suicide data were partially projected due to delays in legal process and data collection. Although NCISH projections are consistently closely correlated with actual numbers with 0 to 5% margin of errors (42) and sensitivity analyses restricted to earlier years were conducted, some residual bias cannot be ruled out.”

5) Differences in suicide data sources may affect results. Mental health patient suicide data (NCISH) and general population suicide data (ONS/NRS) are collected using different methodologies and case definitions, potentially limiting direct comparison between these outcomes

Response: There are no differences in suicide data sources; NCISH cases are simply a subset of ONS/NRS cases. ONS/NRS establishes whether a death is a suicide; NCISH then establishes mental health service contact for those cases. NCISH does not independently ascertain cause of death. This is explained in the Methods section, lines 157-159 and hopefully this is clear.
